# Under the Threat of an Epidemic: People with Higher Subjective Socioeconomic Status Show More Unethical Behaviors

**DOI:** 10.3390/ijerph18063170

**Published:** 2021-03-19

**Authors:** Ting Wang, Xue Wang, Tonglin Jiang, Shiyao Wang, Zhansheng Chen

**Affiliations:** 1 School of Psychological and Cognitive Sciences and Beijing Key Laboratory of Behavior and Mental Health, Peking University, Beijing 100871, China; tingw923@163.com; 2 Marketing Department, The Chinese University of Hong Kong, Hong Kong 999077, China; wangx@link.cuhk.edu.hk; 3 Department of Psychology, The University of Hong Kong, Hong Kong 999077, China; msyaoyao@connect.hku.hk (S.W.); chenz@hku.hk (Z.C.)

**Keywords:** epidemic, social class, socioeconomic status, unethical behaviors

## Abstract

This research focused on the psychological impact of an epidemic. We conducted a cross-sectional survey and two empirical experiments to examine how an epidemic would influence unethical behaviors and how the effect differs in people of different subjective socioeconomic statuses. These studies consistently demonstrated that subjective socioeconomic status moderates the relationship between an epidemic and unethical behaviors. Specifically, the perceived severity of an epidemic positively predicts the unethical behaviors of people with a high socioeconomic status, but it does not predict the unethical behaviors of people with a low socioeconomic status. These findings elucidate the effects of epidemics and bring theoretical and practical implications.

## 1. Introduction

As a sudden outbreak of a deadly virus, an epidemic occurs when the virus spreads rapidly and infects many people quickly through direct person-to-person or secondary contact. An epidemic leaves healthy people vulnerable to risk and has a high mortality rate. For example, the influenza A (H1N1) pdm09 virus, first detected in the United States in the spring of 2009, infected an estimated 100,500,000 people and caused more than 12,000 deaths worldwide [1]. The epidemic also affected psychological health wholesale [2]. Fear, anxiety, depression, distress, and even post-traumatic stress disorder symptoms appeared in infected patients, health-care workers, and the public [3,4,5,6,7]. Besides the heavy toll on physical and psychological health, an epidemic’s rapid spread always overwhelms the whole society. For example, the outbreak of novel coronavirus disease 2019 (COVID-19) has spread across the globe, and the International Health Regulations Emergency Committee of the World Health Organization (WHO) declared the COVID-19 outbreak a global pandemic [8]. It spreads easily and lacks an efficient vaccine; thus, travel restrictions and business closings constitute common prevention measures, which have severely impacted domestic and global economies. 

The health and economic costs of epidemics appear clear, but no research has examined the ethical costs. In our current research, we aimed to fill this gap by examining whether, as an epidemic’s severity increases, people become more likely to display unethical behaviors. Moreover, we explored whether people’s subjective socioeconomic status (SES)—their relative standing in a societal hierarchy regarding wealth, education, and occupation [9,10,11]—would condition the effect of an epidemic’s perceived severity on unethical behaviors.

### 1.1. Epidemics and Unethical Behaviors

People behave unethically toward others to compensate for their personal suffering [12]. For example, ostracism deprives people of valuable connections and benefits. Once ostracized, people feel entitled and thus behave dishonestly to compensate for their suffering [13]. Similarly, when allowed to distribute rewards, previously under-rewarded people are more likely to keep more undeserved rewards at the expense of others’ interests [14]. As an epidemic worsens, the rapid spread and high death toll leave people at higher risk of infection or even death. In addition, people will more likely experience anxiety, depression, and stress—common affects during an epidemic [3]. Beyond the physical and psychological suffering, societal dysfunction and an uncertain future always overwhelm people’s lives. As an epidemic’s perceived severity intensifies, suffering increases dramatically and so do people’s unethical behaviors.

Moreover, people struggle to compromise in the conflict between benefiting from unethical behavior and maintaining a positive self-image [15]. Gaining benefits meets short-term desires, and maintaining a positive self-concept meets a long-term one. People behave unethically when a short-term, self-benefiting goal overrides the long-term goal of maintaining a positive self-image [16]. As an epidemic’s perceived severity increases, people’s lives become more unstable and uncertain. A novel, acute fear of the unknown then arises. The unpredictable future shifts people’s attentions to current needs, even at the expense of long-term goals. Hence, people are more likely to behave unethically as the epidemic’s perceived severity increases. 

Previous studies have shown that psychological distress associates with unethical behaviors [17,18,19,20]. For example, anger increases unethical behaviors, but guilt reduces unethical behaviors [21]. Anxiety and disgust also trigger unethical behaviors [20,22,23,24]. People frequently report psychological distress during epidemics [3]. The threat and uncertainty prompted by an epidemic makes people feel anxious and angry. Becoming a potential victim without doing anything wrong elicits anger, but not guilt. Thus, psychological distress during an epidemic constitutes another possible trigger for unethical behaviors. As the epidemic’s perceived severity increases, people feel greater psychological distress and become more likely to behave unethically.

In summary, we hypothesized that epidemics influence people’s unethical behaviors. An epidemic turns people into potential victims of a deadly disease without any personal wrongdoing, making individuals’ lives unstable and uncertain. During an epidemic, people may see their suffering as unjust and undeserved, thus they focus on short-term desires and feel psychologically distressed. Undeserved suffering, an uncertain future, and psychological distress elicit unethical behaviors during epidemics. Therefore, more frequent unethical behaviors will likely appear as the epidemic’s perceived severity increases. However, an epidemic’s threat does not affect all people uniformly. People with different SESs showed different unethical behaviors during an epidemic.

### 1.2. The Moderating Role of Subjective SES

Differences in people’s wealth and resources shape their relative social standings, translating into differences in their self-construal, attitudes, and behaviors. Low-SES people have limited resources and cannot afford protections from threats. Thus, low-SES people develop interdependent and connected self-construals, whereas affluence bestows high-SES people with independent and distinctive self-construals [9,25,26,27]. 

Low-SES people have lives replete with scarcity, uncertainty, and unpredictability. They feel less personal influence and control [28,29,30] and must develop a cooperative strategy to survive. Thus, low-SES people have consistently appeared responsive to social cues [9]. In social interactions, they showed greater social interest [31], social-oriented emotions [32], and cognizance of others’ emotions [33]. In contrast, abundant resources enable high-SES people to feel a greater sense of control, helping them develop a self-reliant strategy and become more likely to exert influence according to their preferences [30,34,35,36]. They become more self-oriented and less sensitive to others’ emotions and needs [31,33,37].

Compared to low-SES people, high-SES people display greater self-beneficial tendencies [38]. A cross-sectional study found high-SES people were more likely to engage in online shaming compared to low-SES people [39]. Similarly, Piff et al. [37] found high-SES people were more likely to cut off other vehicles or pedestrians while driving, make unethical decisions, take candies meant for nearby children, lie in negotiations, cheat to win a cash prize, and engage in unethical behaviors at work. In sharp contrast, low-SES people showed concern for others’ welfare and were more likely to engage in prosocial behavior. Piff et al. [40] found that low-SES people gave more credit to others in a dictator game, allocated more income to charitable donations, left more points for partners in trust games, and chose long-duration tasks for themselves instead of for their partners. 

Differences in resources and life stability further shape people’s reactions to threats. We focused on one socioecological factor—the epidemic—and examined whether people from different SES backgrounds demonstrated unethical behaviors differently under the epidemic’s threat. High-SES people have more resources and may feel more loss than low-SES people do when society rapidly becomes semi-paralyzed by an epidemic. By contrast, low-SES people face daily life challenges and have less to lose during an epidemic. For example, company owners have more to lose than employees (who lose their jobs) if the company closes. Similarly, people with higher incomes are more likely to have greater salary losses due to closing workplaces resulting from the lockdown during an epidemic. Furthermore, high-SES people are more psychologically entitled than their low-SES counterparts [41]. Low-SES people strive for wealth, but high-SES people want to preserve their privileged statuses [42,43]. Regarding societal instability, high-SES people have more fear of losing their familiar privileged status [43]. Thus, compared to low-SES people, high-SES people may feel they have more to lose and appear more sensitive to severe threats. Jetten et al. [43] investigated whether the 2008 global financial crisis affected low- or high-SES people more. In conditions with unstable (vs. stable) economic prospects, high-SES individuals became more likely to oppose immigration, whereas economic instability did not affect low-SES people’s opposition to immigration, suggesting that high-SES people might be more subjectively sensitive to the threat brought by immigration.

An epidemic endangers people’s survival even if they do nothing wrong. No one can totally avoid an epidemic threat. Similar to low-SES people, high-SES people face infection, death, and other losses from social dysfunction. Moreover, high-SES people are more self-focused and psychologically entitled [41]. Compared to low-SES people, high-SES people fear losing privileged positions that they strive to maintain, so they feel more deprived as the epidemic’s perceived severity increases. 

Epidemics create unpredictability and instability. Low-SES people experience chronic uncertainty and instability, but high-SES people are not conditioned to cope with unexpected or abnormal states. High-SES people have particular difficulty becoming accustomed to the instability and uncertainty brought by an epidemic. Thus, high-SES people will probably appear shortsighted, prioritizing their immediate self-interest over long-term goals as the epidemic’s perceived severity increases. In contrast, low-SES people already have a lower sense of personal control in their daily lives. Thus, high-SES people feel thwarted by the loss of control under an epidemic. Thus, as the epidemic’s perceived severity increases, high-SES people experience more psychological distress associated with suffering.

People behave unethically to compensate for loss, to meet current self-interest needs, and to alleviate psychological distress [12,13,16,44,45,46]. Moreover, high-SES people feel that they have more to lose and a strong motivation to maintain their privileged statuses [43], which makes them more subjectively sensitive to epidemic threats compared to low-SES people. Thus, as an epidemic’s perceived severity increases, high-SES people will likely show more frequent unethical behaviors to meet their current needs, to compensate for their losses of privileged positions and their weakened sense of control, and to relieve associated distress. In contrast, low-SES people would be less psychologically affected. Thus, we hypothesized that, relative to low-SES people, high-SES people will likely show more frequent unethical behaviors as an epidemic’s severity increases. 

### 1.3. The Current Research

We conducted three studies to examine the aforementioned hypotheses. In Study 1, participants from China took a cross-sectional survey about the COVID-19 virus in February 2020, when COVID-19 was spreading rapidly throughout the country. We measured the epidemic’s perceived severity and the participants’ unethical intentions and subjective SESs. In Study 2, people from the United States participated in an empirical experiment. Epidemic severity was manipulated via an imagination task. We measured unethical intentions and subjective SESs. In Study 3, we employed a different sample (i.e., Chinese) and measured actual unethical behaviors. We expected that high-SES people would demonstrate more frequent unethical behaviors when the epidemic’s perceived severity was high (vs. low) and that the perceived epidemic severity would not impact low-SES people’s unethical behaviors.

## 2. Study 1

In Study 1, we conducted a cross-sectional investigation in China when the COVID-19 virus was rapidly spreading across the country. We examined the covariation of perceived epidemic severity and unethical behaviors, as well as the moderating role of subjective SESs.

### 2.1. Materials and Methods

#### 2.1.1. Participants 

Based on Monte Carlo simulations, Schönbrodt and Perugini [47] recommended using a typical sample of approximately 250 for stable estimates. We tested 651 participants in mainland China (217 men, 434 women; M age = 27.42 years, SD age = 7.30 years), remunerating them with RMB 2.5 (approximately USD 0.36). They all passed two attention check questions (i.e., “Please choose 4”; “Please choose moderately agree”) [48]. We asked participants to complete an online survey about COVID-19 carefully. We oversampled because this study comprised part of a large-scale survey with measures not reported in this study.

#### 2.1.2. Procedure and Materials 

Participants first reported their demographic information. We measured their subjective SES using the MacArthur Scale of Subjective Socioeconomic Status. Then, we asked participants to select a position on a 10-rung ladder to represent their perceived standings in society. Higher scores indicated higher levels of subjective SES.

We assessed perceived epidemic severity via six items. The items were: “How serious you think the COVID-19 is in the place you live?” “How serious do you think the COVID-19 virus is in the area you live?” “How serious do you think the COVID-19 virus is in your neighborhood?” “How likely do you think your neighbors are to be affected?” “How likely do you think your family members are to be affected?” and “How likely do you think you are to be affected?” (1 = not at all, 7 = very much so). We averaged the responses to form a composite (α = 0.92). Higher scores indicated greater perceived epidemic severity.

We assessed unethical behaviors using two measures, adapting the first from the Propensity to Engage in Unethical Behaviors scale [49]. We used five vignettes instead of the whole scale to keep the survey short. The items chosen were: “Use office supplies (paper, pen), Xerox machine, and stamps for personal purposes”, “Take no action when customers shoplift”, “Borrow RMB 20 from a cash register overnight without asking”, “Receive gifts, money, and loans (bribery) from others due to your position and power”, and “Make more money by deliberately not letting clients know about their benefits”. Participants reported how likely they would be to behave as depicted if they were in each of the above situations, (1 = very unlikely, 7 = very likely). Responses to the five vignettes were averaged to index unethical intention scenarios (α = 0.89).

The second measure included three self-composed items: “I would falsify my work experience during a job application”, “I would keep the money if I picked up a wallet”, and “I would keep silent if I found that my mobile payment was not successful and the salesperson was not aware of it”. Similarly, we asked participants to indicate the likelihood they would behave in the way depicted in these vignettes on a 7-point scale (1 = very unlikely to 7 = very likely). Responses to the three items were averaged to index self-composed unethical intentions (α = 0.81).

### 2.2. Results and Discussion

Perceived epidemic severity correlated significantly with unethical intention scenarios, r (651) = 0.24, *p* < 0.001, 95% CI [0.16, 0.31], and self-composed unethical intentions, r (651) = 0.23, *p* < 0.001, 95% CI [0.16, 0.31]. Unethical intention scenarios positively associated with self-composed unethical intentions, r (651) = 0.82, *p* < 0.001, 95% CI [0.80, 0.85]. That is, people who perceived the epidemic as more severe had more unethical intentions.

**Figure 1:**
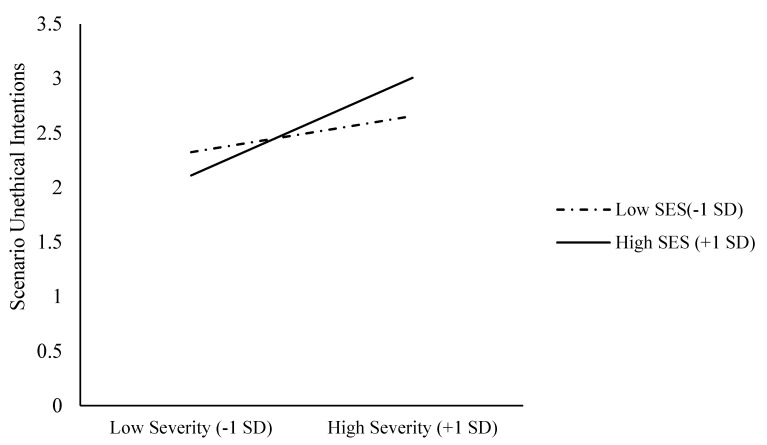
Unethical intention scenarios as functions of epidemic severity and SES (Study 1).

Next, we tested whether SES moderated the relationship between epidemic severity and unethical intentions. We standardized the epidemic severity and SES scores and conducted two bootstrapping analyses (5000 iterations) via Hayes’s (2013) SPSS macro PROCESS. First, we entered unethical intention scenarios as the dependent variable, the perceived epidemic severity as the independent variable, and the SES as a moderator into Model 1 [50]. The main effect of the epidemic’s perceived severity on unethical intention scenarios was significant, b = 0.31, SE = 0.06, t = 5.04, *p* < 0.001, 95% CI [0.19, 0.43]. The main effect of SES on unethical intention scenarios was not significant, b = 0.03, SE = 0.06, t = 5.74, *p* = 0.57, 95% CI [−0.08, 0.15]. More importantly, the interaction effect was significant, b = 0.14, SE = 0.05, t = 2.75, *p* = 0.006, 95% CI [0.04, 0.24] (see Figure 1). With a high SES (+1 SD), the epidemic’s perceived severity positively associated with unethical intention scenarios, b = 0.45, SE = 0.07, t = 6.49, *p* < 0.001, 95% CI [0.31, 0.58]. With a low SES (−1 SD), however, the epidemic’s perceived severity did not associate with unethical intention scenarios, b = 0.17, SE = 0.09, t = 1.86, *p* = 0.06, 95% CI [−0.008, 0.34]. When perceived epidemic’s severity was high (+1 SD), SES was positively associated with unethical intention scenarios, b = 0.18, SE = 0.07, t = 2.43, *p* = 0.016, 95% CI [0.03, 0.32]. With low perceived epidemic severity (−1 SD), however, SES did not associate with unethical intention scenarios, b = −0.11, SE = 0.08, t = −1.26, *p* = 0.21, 95% CI [−0.27, 0.06].

**Figure 2:**
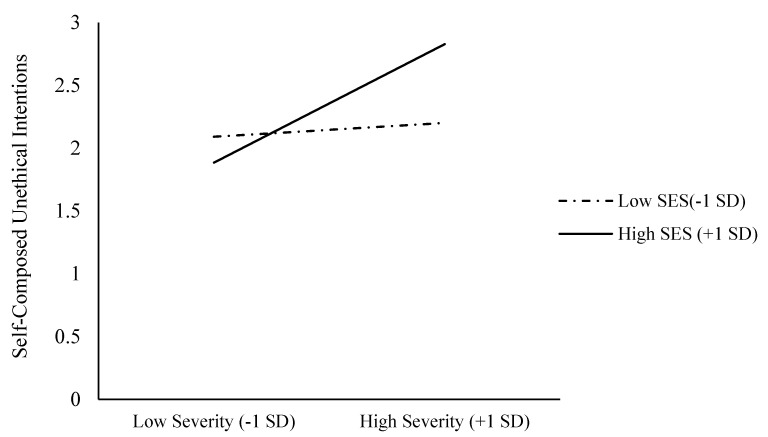
Self-composed unethical intentions as functions of epidemic severity and SES (Study 1).

Similar results appeared when we used self-composed unethical intentions as the dependent variable. Our bootstrapping analysis used 5,000 iterations. The main effect of the epidemic’s perceived severity on self-composed unethical intentions was significant, b = 0.26, SE = 0.06, t = 4.41, *p* < 0.001, 95% CI [0.15, 0.38]. The main effect of SES on self-composed unethical intentions was not significant, b = 0.10, SE = 0.06, t = 1.79, *p* = 0.074, 95% CI [−0.01, 0.22]. Importantly, the interaction effect was significant, b = 0.21, SE = 0.05, t = 4.14, *p* < 0.001, 95% CI [0.11, 0.31] (see Figure 2). With a high SES (+1 SD), the perceived epidemic severity positively associated with self-composed unethical intentions, b = 0.47, SE = 0.07, t = 6.97, *p* < 0.001, 95% CI [0.34, 0.60]. With a low SES (−1 SD), however, perceived epidemic severity did not associate with self-composed unethical intentions, b = 0.06, SE = 0.09, t = 0.64, *p* = 0.53, 95% CI [−0.12, 0.23]. With high perceived epidemic severity (+1 SD), SES positively associated with unethical intention scenarios, b = 0.31, SE = 0.07, t = 4.42, *p* < 0.001, 95% CI [0.17, 0.45]. With low perceived epidemic severity (−1 SD), however, SES did not associate with unethical intention scenarios, b = −0.10, SE = 0.08, t = −1.24, *p* = 0.21, 95% CI [−0.27, 0.06].

Study 1 supported our hypothesis that unethical intentions were positively associated with the perception of epidemic severity. Moreover, SES moderates the relationship between the perceived epidemic severity and unethical intentions. Those who perceive themselves as having high SES display more unethical intentions as epidemics worsen. In contrast, those who perceive themselves as having low SES show no significant relationship between perceived epidemic severity and unethical intentions. Moreover, a higher SES relates to increased unethical intentions only with high perceived epidemic severity. Study 1 was correlational in nature but was limited in providing casual evidence. We addressed this limitation in Study 2.

## 3. Study 2

Study 2 extends Study 1 in two ways. First, we manipulated the perceived epidemic severity to provide causal evidence for our suggested moderation model. Second, we recruited Americans as participants to increase our findings’ generalizability.

### 3.1. Method

#### 3.1.1. Participants

A G*Power analysis showed we needed at least 265 participants to detect a medium effect size (*f* ^2^ = 0.04) with a power of 0.90 (α = 0.05). We tested 333 participants using Amazon’s Mechanical Turk (U.S. residence, human intelligence test approval rate > 0.90%), remunerating them with $0.20 each. We asked participants to do an imagination task and answer some questions. Seven people did not complete the survey, and 45 did not pass the two attention check questions. We included the remaining 281 participants in Study 2 (135 men, 146 women; Mage = 42.15 years, SDage = 13.53 years). 

#### 3.1.2. Procedure and Materials 

Participants were randomly assigned to low- (n = 148) or high-severity (n = 133) conditions. In both conditions, participants were told that NEMRS-834 is one of the deadliest novel coronaviruses and were then asked to imagine its spread as instructed. Participants in the low-severity condition read the following:

Now, please imagine a man living in your neighborhood was infected with NEMRS-834. A total of one hundred and fifty-eight people who had direct and indirect contact with him were identified, but no one became infected. Scientists estimate the infected number will not increase dramatically, even though more people who had indirect contact with him will be identified. Scientists claim that there is no sign showing that NEMRS-834 is spreading in your neighborhood or your city.

Participants in the high-severity condition read the following:

Now, please imagine a man living in your neighborhood got infected with NEMRS-834. Now, 158 people who had direct and indirect contact with him were identified, and all of them got infected. Scientists estimate the infected number will increase dramatically as more people who had indirect contact with him are identified. Scientists claim that NEMRS-834 is spreading in your neighborhood and your city.

They were asked to imagine the scenarios as vividly as possible. After, participants were asked to judge four statements: “How likely you are to be infected?” “How likely are people living nearby to be infected?” “How likely are people living in your city to be infected?” and “How serious is the epidemic?” (1 = not at all, 9 = very much). We averaged the four items’ scores to index perceived severity (α = 0.91). Higher scores indicated greater perceived epidemic severity.

We assessed unethical intentions with the 15-item Propensity to Engage in Unethical Behaviors scale [49]. Sample items include “using office supplies (paper, pen), the Xerox machine, and stamps for personal purposes” and “take no action to stop shoplifting by customers” (1 = very unlikely, 7 = very likely). We averaged the responses to the 15 items to index unethical intention scenarios (α = 0.95). Higher scores indicated higher levels of unethical intentions.

Afterward, participants reported their demographic information. We measured subjective SES the same way as in Study 1. Higher scores indicated higher levels of perceived SES. Finally, we fully debriefed participants and thanked them for their participation.

### 3.2. Results and Discussion

#### 3.2.1. Manipulation Check 

Participants in the high-severity condition (M = 7.38, SD = 1.50) perceived the epidemic as more severe than participants in the low-severity condition, M = 4.76, SD = 1.89, t(275.03) = 12.92, *p* < 0.001, Cohen’s d = 1.54, 95% CI [1.28, 1.81]. We successfully manipulated the perceived epidemic severity.

#### 3.2.2. Unethical Intentions

Participants in the high- (M = 2.42, SD = 1.41) and low-severity (M = 2.22, SD = 1.22) conditions did not differ in unethical intentions, t(262.74) = 1.21, *p* = 0.23, Cohen’s d = 0.14, 95% CI [−0.09, 0.38]. That is, the epidemic’s perceived severity alone did not influence unethical intentions.

#### 3.2.3. Moderation Model 

**Figure 3:**
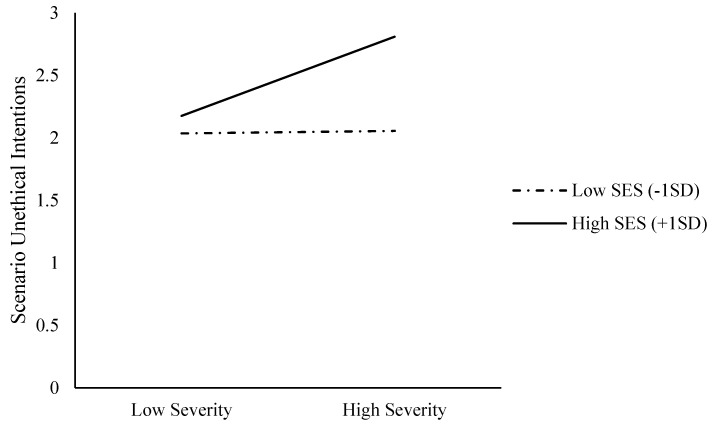
Unethical intention scenarios as functions of epidemic severity and SES (Study 2).

Next, we tested whether SES moderates the relationship between perceived epidemic severity and unethical intentions. We standardized the SES scores and coded the low-severity condition as -1 and the high-severity condition as 1. As in Study 1, we conducted a bootstrapping analysis (5,000 iterations) with Hayes’ (2013) [50] SPSS macro PROCESS. First, we entered unethical intention scenarios as the dependent variable, the perceived epidemic severity as the independent variable, and SES as a moderator into Model 1 [50]. The main effect of the epidemic’s perceived severity on unethical intention scenarios (b = 0.16, SE = 0.07, t = 2.18, *p* = 0.030, 95% CI [0.02, 0.31]) and the SES’s main effect on unethical intention scenarios (b = 0.22, SE = 0.08, t = 2.97, *p* = 0.003, 95% CI [0.08, 0.37]) were significant. More importantly, the interaction effect was significant, b = 0.15, SE = 0.08, t = 2.04, *p* = 0.042, 95% CI [0.005, 0.30] (see Figure 3). When the SES was high (+1 SD), the main effect of perceived epidemic severity on unethical intention scenarios was significant, b = 0.32, SE = 0.11, t = 2.99, *p* = 0.003, 95% CI [0.11, 0.53]. When the SES was low (−1 SD), however, the main effect of perceived epidemic severity on unethical intention scenarios was not significant, b = 0.01, SE = 0.11, t = 0.09, *p* = 0.93, 95% CI [−0.19, 0.22]. When perceived epidemic severity was high (+1), SES positively associated with unethical intention scenarios, b = 0.38, SE = 0.10, t = 3.70, *p* = 0.003, 95% CI [0.18, 0.58]. When the perceived epidemic severity was low (−1), however, SES did not associate with unethical intention scenarios, b = 0.07, SE = 0.11, t = 0.63, *p* = 0.53, 95% CI [−0.15, 0.29].

Consistent with Study 1, these results indicate that high-SES people’s unethical intentions increase as the perceived epidemic severity increases. For low-SES people, however, perceived epidemic severity does not influence unethical intentions. These results also demonstrate that a higher SES associates with increased unethical intentions only when the epidemic’s perceived severity is high. However, Studies 1 and 2 only tested unethical intentions. Study 3 addressed this limitation by testing actual unethical behaviors.

## 4. Study 3

Previous studies consistently found that SES moderates the impact of an epidemic’s perceived severity on unethicality. For high-SES people, unethical intentions increase as the perceived epidemic severity increases. However, perceived epidemic severity does not influence the unethical intentions of low-SES people. As a limitation, previous studies measured unethical behavior with hypothetical scenarios. Importantly, we manipulated the perceived pandemic severity and measured actual unethical behaviors in Study 3. 

### 4.1. Method

#### 4.1.1. Participants

As with Study 2, we needed at least 265 participants to detect a medium effect size (*f* ^2^ = 0.04) with a power of 0.90 (α = 0.05). We recruited 350 participants from the reliable Chinese recruitment platform Credamo, remunerating each of them with RMB 2 (approximately USD 0.30). We excluded 66 participants who failed two attention check questions and retained 284 participants (166 men and 118 women; Mage = 28.46 years, SDage = 5.31 years).

#### 4.1.2. Procedure and Materials

We randomly assigned participants to a high-(n = 142) or low-severity (n = 142) condition. They first finished the same pandemic severity manipulation as in Study 2. We used Brislin’s (1980) procedures to translate and back translate all materials and measures from English to Chinese. We asked participants to imagine scenarios as vividly as possible. Afterwards, they judged three statements intended to check the manipulation effectiveness (e.g., “How likely you are to be infected?”; 1 = not at all to 9 = very much so). We averaged the three items’ scores to index severity (α = 0.91). Higher scores indicated greater perceived epidemic severity.

Participants then joined a task intended to measure their real unethical behavior. Participants read a cover story that researchers had already conducted eight studies about COVID-19 over the past months and that their study comprised the ninth one. The researchers wanted to remunerate people who participated in five or more studies. Specifically, people who participated five times received RMB 5 (approximately USD 0.75), those who participated six times received RMB 10 (approximately $1.50), seven times received RMB 15 (approximately $2.25), eight times received RMB 20 (approximately $3.00), and nine times received RMB 25 (approximately $3.75). We asked participants to report the number of studies in which they had participated (including the current one). They were told they could check the feedback e-mail from each study they had participated in if they could not remember the times clearly. In fact, researchers had not conducted multiple studies and did not send out any feedback e-mail. Therefore, the correct number of studies the participants had joined should be one. Larger numbers reported by participants indicated higher levels of unethicality. The unethical behaviors were coded as follows: 0 (fewer than 5 times), 1 (5 times), 2 (6 times), 3 (7 times), 4 (8 times), and 5 (9 times).

Afterward, participants reported their demographic information. We assessed SESs via the same measure as in Study 1. Higher scores indicated higher levels of perceived SES. Finally, we fully debriefed and thanked participants. All participants then went through a lucky draw, where they could earn an extra reward of RMB 20 (approximately $3.00).

### 4.2. Results and Discussion

#### 4.2.1. Manipulation Check

Participants in the high-severity condition (M = 7.71, SD = 1.91) perceived more severity than did participants in the low-severity condition, M = 4.61, SD = 2.60, t (282) = 11.46, *p* < 0.001, Cohen’s d = 0.68, 95% CI [0.55, 0.81]. This confirms the severity manipulation’s effectiveness.

#### 4.2.2. Unethical Behaviors

Nonsignificant differences of unethical behaviors existed between participants in the high-(M = 1.10, SD = 1.63) and low-severity conditions (M = 0.96, SD = 1.50), t (282) = 0.75, *p* = 0.45, Cohen’s d = 0.04, 95% CI [−0.07, 0.16]. Thus, the perceived severity of the epidemic alone did not affect unethical behaviors.

#### 4.2.3. Moderation Model 

**Figure 4:**
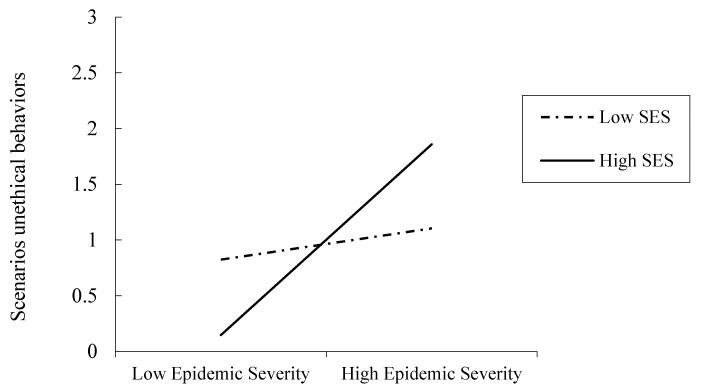
Unethical behavior scenarios as functions of epidemic severity and SES (Study 3).

We examined whether SES would moderate the epidemic severity manipulation’s effect on unethical behaviors. As in Study 2, we employed Hayes’s PROCESS Model 1 [50] and used 5000 samples to test the moderation. First, we entered unethical behaviors as the dependent variable, the epidemic severity’s effect code (high = 1, low = −1) as the independent variable, and standardized SES as a moderator. A significant main effect of SES appeared (b = 0.19, SE = 0.09, t = 2.12, *p* = 0.034, 95% CI [0.01, 0.38]). A significant main effect of SES appeared, b = 0.19, SE = 0.09, t = 2.12, *p* = 0.034, 95% CI [0.01, 0.38]. A nonsignificant main effect of epidemic severity appeared, b = 0.14, SE = 0.18, t = 0.766, *p* = 0.44, 95% CI [−0.22, 0.51]. More importantly, the interaction effect was marginally significant, b = 0.35, SE = 0.18, t = 1.92, *p* = 0.056, 95% CI [−0.009, 0.72] (see Figure 4). When the SES was high (+1 SD), perceived epidemic severity positively (yet marginally) predicted unethical behavior, b = 0.50, SE = 0.26, t = 1.90, *p* = 0.058, 95% CI [−0.02, 1.02]; when the SES was low (−1 SD), perceived epidemic severity did not predict unethical behavior, b = −0.22, SE = 0.26, t = −0.82, *p* = 0.411, 95% CI [−0.73, 0.30]. When perceived epidemic severity was high (+1), SES positively associated with unethical behavior scenarios, b = 0.38, SE = 0.14, t = 2.71, *p* = 0.007, 95% CI [0.10, 0.65]. When perceived epidemic severity was low (−1), however, SES did not associate with unethical behavior scenarios, b = 0.01, SE = 0.12, t = 0.15, *p* = 0.88, 95% CI [−0.23, 0.26].

Thus, consistent with results from Studies 1 and 2, we found that perceived epidemic severity positively predicts the unethical behaviors of high-SES people but not those of low-SES people.

## 5. Discussion

An epidemic is a sudden outbreak of an infectious virus for which little or no immunity exists in the human population. Epidemics happen irregularly and rarely but severely impact societies. The heavy tolls on the public’s physical and psychological health and on the domestic and global economies appear clear, but no research exists to investigate the ethical costs of epidemics. Using the complementary methodologies of cross-sectional surveys and empirical experiments, our research fills this gap. We have found that the perceived severity of epidemic predicts unethical behaviors and that subjective SESs condition this effect. Specifically, we have found that the perceived severity of an epidemic predicts unethical behaviors among high-SES people but not among low-SES people. 

### 5.1. Research Implications

Our findings are consistent in Chinese and US samples. Chinese participants experienced the spread of COVID-19 during our correlational data collection, demonstrating our research’s ecological validity and generalizability. These findings have implications for the socioecological environment’s psychology, which focuses on how socioecological factors impact human behaviors [23]. 

The association between an epidemic’s perceived severity and unethical behaviors differs with the SES. Specifically, the unethical behaviors of high-SES people increase as the epidemic’s perceived severity increases. However, low-SES people generally exhibit fewer unethical behaviors, which remains unaffected by the epidemic’s perceived severity. When an extremely threatening case suddenly appears, high-SES people are more subjectively sensitive to the epidemic’s threat. As the epidemic worsens, high-SES people are more likely to sense loss, uncertainty, and associated distress than low-SES people are, which further increases unethical behaviors. These results align with previous literature suggesting that high-SES people are more self-focused, entitled, and sensitive to threats than low-SES people are [43]. 

Notably, we do not argue against low-SES people having harder lives during an epidemic or even that an epidemic might disproportionately impact low-SES people because they have few recourses to cope with sudden outbreaks. We suggest that a sudden epidemic affects the psychological characteristics of high-SES people, making them more sensitive to an epidemic’s threat—at least psychologically—because high-SES people feel that they have more to lose and that they have a strong motivation to preserve their privileged statuses [43,51]. This motivation leads to more frequent unethical behaviors. 

Moreover, with low perceived epidemic severity, low- and high-SES people have similar levels of unethical behavior. With high perceived epidemic severity, however, unethical behaviors increase as people’s perceived SES increases. These findings contribute to SES research. Some recent inconsistency and debate exist about how low- and high-SES people would behave during epidemics. For example, high-SES people have appeared more likely to make charitable donations, do volunteer work, and show trust in an economic game [52]. Furthermore, low-SES people have behaved aggressively [53]. Considering boundary conditions could resolve this inconsistency. For example, Kraus and Callaghan [54] suggested that the anonymity of prosociality influences the relationship between SES and prosocial behavior. They found that high-SES people appeared more prosocial in public than they did in private, whereas low-SES people showed the reverse pattern [54]. Dubois et al. [38] found that low- and high-SES people both behaved unethically. They further clarified that high-SES people behaved unethically to benefit themselves, but low-SES people behaved unethically to benefit others [38]. Our research expands previous SES research by suggesting that epidemic contexts condition the relationship between SES and unethical behavior. A higher SES predicts more frequent unethical behavior only with a high perceived epidemic severity.

### 5.2. Policy Implications

During an epidemic, the heavy tolls on the public’s physical and psychological health and on the domestic and global economies draw great attention. Our research suggests that, beyond the health and economic costs, attention should be paid to the ethical costs. Unethical behavior is a risk factor for societal stability and development. Lowering the ethical costs brought by the epidemic is another important task for the epidemic control and prevention. 

In addition, high-SES people are more likely to conduct unethical behaviors as the epidemic severity increases, thus policy makers should take initiatives to cope with this issue. For example, besides material and psychological help for low- and high-SES people, social media, psychological counseling, community service, and other means could be adopted to guide high-SES people’s psychological states to lower the ethical costs of an epidemic. For example, the community could encourage high-SES people to participate in mutual aid activities to make them realize the importance and benefits of social support and interdependence and to reduce the feelings of psychological entitlement. 

### 5.3. Limitations and Future Directions

First, in Studies 1 and 2, we measured unethicality through self-reported scales. Although we addressed this limitation by measuring actual unethical behaviors in Study 3, we should be cautious of the generalizability of our findings about relationships between epidemic severity and unethical behaviors. Future research should further explore diverse and valid unethical behavior measures.

Second, we began our investigation in early February 2020, when COVID-19 was rapidly spreading within China and was deemed an epidemic. In Study 1, we studied the epidemic severity of COVID-19. In Studies 2 and 3, to manipulate epidemic severity, we used fictitious epidemic materials. Our findings shed light on the relationship between epidemic severity and unethical behaviors, but we could not simply conclude that the severity of COVID-19 has increased unethical behaviors. COVID-19 has become a global pandemic as time has continued, thus the relationship between COVID-19 severity and unethical behaviors might become more complicated. Future research could investigate the dynamic relationship between COVID-19 severity and unethical behaviors during the development of COVID-19.

Third, in current research, we found epidemic severity predicts unethical behaviors for people with high SES rather than low SES. Future research could also test the underlying mechanism. As discussed, we suggest that the relative sense of deprivation, instability, uncertainty, and the associated psychological distress are potential mediators and could explain why the epidemic’s perceived severity is associated with unethical behaviors for high-SES people. 

Fourth, in Studies 2 and 3, we did not find a significant effect of perceived epidemic severity on unethical intentions. Two possible explanations exist. First, the main effect of the epidemic’s severity may be conditioned by SES. Second, we successfully manipulated the epidemic severity in Studies 2 and 3 via an imagination method. However, the effect may be weaker than it is in the real epidemic context used in Study 1, thus showing a nonsignificant effect for perceived epidemic severity. Future research could explore a robust method to manipulate the perceived epidemic severity ethically.

## 6. Conclusions

In conclusion, we found that epidemics impact morality and that high-SES people are more likely to engage in unethical behavior as an epidemic’s perceived severity increases. In addition to health and economic costs, the moral costs of epidemics are worth attention. Morality is another important aspect for policy makers to consider when battling epidemics. 

## Data Availability

The data presented in this study are available on request from the corresponding author.
